# Cultural Adaptation of Sniffin’ Sticks Smell Identification Test: The Malaysian Version

**DOI:** 10.22038/ijorl.2019.34346.2138

**Published:** 2020-07

**Authors:** Lum Sai-Guan, Salina Husain, Farah-Dayana Zahedi, Norfazilah Ahmad, Balwant-Singh Gendeh

**Affiliations:** 1 *Department of Otorhinolaryngology – Head and Neck Surgery, University Kebangsaan Malaysia Medical Centre, Kuala Lumpur, Malaysia. *; 2 *Department of Community Health, Faculty of Medicine, University Kebangsaan Malaysia Medical Centre, Kuala Lumpur, Malaysia.*

**Keywords:** Cultural adaptation, Olfaction, Smell, Smell Identification, Sniffin’ Sticks

## Abstract

**Introduction:**

Sniffin’ Sticks smell identification test is a tool used for evaluation of olfactory function but the results are culture-dependent. It relies on the subject’s familiarity to the odorant and descriptors. This study aims to develop the Malaysian version of Sniffin’ Sticks smell identification test suitable for local population usage.

**Materials and Methods:**

The odorant descriptors and distractors of the original version of Sniffin’ Sticks were translated into Malay language. It was then tested for familiarity and identifiability in 30 normosmic subjects. The descriptors were replaced until the familiarity of all descriptors and identification rates of odorants achieved ≥ 70%. The validity of the new cultural-adapted version was tested in 60 hypo-anosmic subjects and 60 normosmic subjects with Student t-test. The test-retest reliability was evaluated after two weeks with interclass correlation.

**Results:**

Two odorant descriptors and nine distractors achieved familiarity <70% (13.3% - 66.7%) and were replaced. Another three culturally inappropriate distractors were also replaced. The mean score among the healthy subjects was significantly higher than the subject with smell dysfunction [13.7 (1.12) and 7.3 (3.42); t = 7.24 (df = 34.23), P<0.001]. The coefficient of correlation (r) between test and retest scores was 0.93 (P<0.001).

**Conclusion:**

The cultural adapted Malaysian version of Sniffin’ Sticks smell identification test is valid and has high test-retest reliability. This is the first smell identification test validated in Malaysia. It is effective for evaluation of olfactory function in local population.

## Introduction

Olfaction is one of the major human senses. Smell can influence our mood, cognition, and behaviour. Human olfactory functions were related to ingestion, behaviour to avoid environmental hazards and social communication (1). Impairment of the olfactory function has significant consequences for health, safety and quality of life. 

Medical practitioners often undervalue olfactory impairment, compared to other sensory loss, such as visual or hearing deficit. Quite commonly the patients are also unaware of their smell dysfunction (2). Smell identification test is an important tool for the clinical evaluation of olfaction, but the results seem to be culture-dependent (3). Smells that are familiar in European countries may not be familiar to Asian populations. Therefore, it cannot be used for direct comparison of olfactory sensitivity between peoples of different cultural background. 

The Sniffin’ Sticks test battery is a psychophysical test for assessment of olfactory function using a pen-like odour dispensing devices. It is one of the commonest olfactory tests used in clinical setting worldwide especially in European countries. It was initially developed and validated in Germany (4,5). Its test-retest reliability and validity has been well established and normative data has been published in Northern Europe (6-8). The usefulness of Sniffin’ Sticks has also been proven in assessing olfaction for various countries and populations e.g. Australia, Greece, Holland, Italy, Portugal, Great Britain, Romania, Egypt, Turkey, Taiwan, Sri Lanka, Korea and Brazil (9-23). 

The Sniffin’ Sticks consists of three subtests, namely smell threshold, smell discrimination, and smell identification. To perform all three subtests is time consuming and not applicable in all centres. Study had suggested that the individual subtest could be used separately to monitor olfactory function with high test-retest reliability (24). Smell identification component appears to be very sensitive in early diagnosis of neurodegenerative disease such as Parkinson’s disease, as olfactory impairment often precedes the onset of motor symptoms. It is a reliable test, simpler and less time consuming compared to other more extensive tests. The result of Sniffin’ Sticks smell identification test (SS-SIT) is affected by cultural differences because it relies on the subject’s familiarity to the test odorants and descriptors. Cultural adaptation is therefore necessary in countries with different cultural background before the test can be used (10,13), Using a non-adaptation version of the SS-SIT can potentially misdiagnose hyposmia in a subject with normal sense of smell (10).

Currently there is no standard method to gauge olfactory dysfunction in local clinical setting. Most of the time otorhinolaryngologists depend on patient’s subjective report of the presence of any smell disorder. Sniffin’ Sticks smell identification test therefore allow for quantitative measurement of smell deficit, rather than subjective description. We study the cultural adaptation of the SS-SIT, and to validate its applicability in Malaysian population. To our knowledge, this is the first smell identification test validated in Malaysia. Not only in Malaysia, the validated test can be used in other regions for Malaysian living abroad or other population sharing the same language and culture. Besides, it can be a valuable reference for similar work in Asian countries.

## Materials and Methods

A cross sectional study was conducted from May 2016 to April 2017 in Otorhinolaryngology clinic of UKMMC, Kuala Lumpur, Malaysia. Ethical approval for the study was obtained from the Research Ethics Committee of UKMMC (UKM 1.5.3.5/244/FF-2015-394). The study was performed in accordance with the Declaration of Helsinki for research on human subjects. Informed consent was obtained from all subjects. The subjects with reduced sense of smell were recruited from patients attending otorhinolaryngology clinic. 

Exclusion criteria were subject less than 18 year-old, underlying neurodegenerative disorder e.g. Alzheimer’s disease and Parkinson’s disease, neuropsychiatric disorder e.g. schizophrenia, pregnancy and recent upper respiratory tract infection within two weeks. Healthy subjects with normal sense of smell were invited to participate the study voluntarily from the hospital staffs or family members of the patients. 

Exclusion criteria were subject less than 18 year-old, underlying nasal pathology, previous nasal surgery, previous severe head trauma, pregnancy and recent upper respiratory tract infection within two weeks. Nasal endoscopy was performed to exclude sinonasal pathology. The smell identification test were performed using sixteen items Sniffin’ Sticks (Burghart Messtechnik, Germany), which consist of 16 reusable pens as applicator of different odorants. 

They are felt-tip pens of 14 cm long and 1.3 cm in diameter, with a tampon filled with 4 ml liquid odorants dissolved in propylene glycol. The test procedure followed the described standard methodology (5). 

It was carried out in a properly ventilated room with the use of odourless gloves. The subjects should neither have eaten nor drunk anything other than plain water 15 minutes prior to the test. This rule extends also to smoking, and the use of nasal topical medication or chewing gum. The subjects were presented with 16 different odorants, with the tip of the pen placed approximately 2 cm in front of nostrils for 2 seconds.

The interval between odour presentations is 20 seconds. Using a multiple forced-choice design, the subjects identify the correct odorant from a list of four descriptors that includes one correct answer and three distractors. One mark will be given for each correctly identified odorant, with total score ranges from 0 to 16. Score ≥12 is considered normosmia, while score <12 is considered hyposmia. The SS-SIT underwent four phases before the final usage. 

**Phase 1: Translation procedure**

The exact translation of the SS-SIT odorant descriptors and distractors were done using the established forward-backward procedure. Two independent bilingual (English and Malay language) health professionals performed translation from English to Malay language. Two different bilingual health professionals then translated the provisional Malaysian version back into English language.

It was not only a word-for-word literal translation, but the conceptual equivalence was determined. Discrepancies were discussed and the process was iterated until a satisfactory version is reached (i.e. the translated Malay language version). The final version was comparable to the original version. 

*Phase 2: Assessment of familiarity of odour descriptors and identifiability of test odorants in the translated version*

A total of 30 normosmic volunteers (13 male, 17 female; mean age, 35.7 [8.6] years; range 23-54) were recruited and asked to identify any non-familiar term from the list of descriptors. The familiarity of each item was expressed in percentage. 

The items with percentage lower than 70% were considered as not acceptable and were replaced by clearer or more familiar terms for better recognition. Then the same subjects were presented with the 16 different test odorants and asked to name the odour from a list of four odour descriptors for each pen. The identifiability for each odour was reported as percentage of correct identification from the list of four descriptors. If any of the test odorant achieved identification rate less than 70%, the distractors were replaced by item with greater contrast until all test odorants achieved identification rate ≥70% (i.e. the cultural adapted version). 

**Phase 3: Evaluation of construct validity using the cultural adapted version**

The cultural adapted version was evaluated in two groups of subjects consisted of 60 subjects each, one group with reduced sense of smell (the hypo-anosmic group; 30 male, 30 female; mean age, 50.5 [17.4] years; range, 18-78) and another group with normal sense of smell (the normosmic group; 24 male, 36 female; mean age, 31.9 [8.7] years; range, 18-54). The mean score was obtained and compared between the two groups. 

*Phase 4: Evaluation of test-retest reliability*

The repeatability of the adapted version of Sniffin’ Sticks was examined by repeating the test on the same group of subjects in phase 3, at least two weeks apart during follow-up. The mean score of the Sniffin’ Sticks smell identification test between the normosmic and the hypo-anosmic group was compared using Student t-test. The test-retest reliability was evaluated by comparing the test and retest mean scores with interclass correlation. The results were analysed using SPSS 22.0 for Windows (SPSS Inc., Chicago, IL). The level of significance was set at *p*<0.05 (95% confidence interval).

## Results

**Phase 1 and 2: Translation procedure and assessment of familiarity of odour descriptors and test odorants**

The translated version and the result of the familiarity are shown in Table 1. Two test odorants had identification less than 70%, namely ‘licorice’ (13.3%) and ‘turpentin’ (36.7%). The original odorants contained within the sticks were unchanged but the descriptors were replaced by terms more familiar to Malaysian, namely ‘licorice’ by ‘jintan manis’ and ‘turpentin’ by ‘pencair cat thinner’ that have the almost similar smell. 

**Table 1:** The translated version of Sniffin’s Sticks odorants and distractors, and its familiarity shown in percentage

**Card**	**Odorant**	**%**	**Distractor 1**	**%**	**Distractor 2**	**%**	**Distractor 3**	**%**
1	Orange (Oren)	100.0	Blackberry (Buah beri hitam)	43.3	Strawberry (Strawberi)	90.0	Pineapple (Nenas)	96.7
2	Leather (Beg kulit)	96.7	Smoke (Asap)	100.0	Glue (Gam)	100.0	Grass (Rumput)	96.7
3	Cinnamon (Kayu manis)	90.0	Honey (Madu)	100.0	Vanilla (Vanila)	90.0	Chocolate (Coklat)	100.0
4	Peppermint (Pudina)	86.7	Chive (Daun kucai)	50.0	Fir (Pokok cemara)	10.0	Onion (Bawang merah)	96.7
5	Banana (Pisang)	100.0	Coconut (Kelapa)	100.0	Walnut (Kacang walnut)	66.7	Cherry (Buah ceri)	80.0
6	Lemon (Lemon)	100.0	Peach (Buah pic)	83.3	Apple (Epal)	96.7	Grapefruit (Limau gedang)	63.3
7	Licorice (Licorice)	13.3	Cherry (Buah ceri)	80.0	Spearmint (Pudina)	86.7	Cookie (Biskut)	96.7
8	Turpentine (Turpentin)	36.7	Mustard (Mustard)	63.3	Rubber (Getah)	93.3	Menthol (Mentol)	73.3
9	Garlic (Bawang putih)	100.0	Onion (Bawang merah)	96.7	Sauerkraut (Acar kobis)	53.3	Carrot (Lobak)	96.7
10	Coffee (Kopi)	100.0	Cigarette (Rokok)	96.7	Wine (Wain)	NT	Candle smoke (Asap lilin)	96.7
11	Apple (Epal)	96.7	Melon (Tembikai)	100.0	Peach (Buah pic)	83.3	Orange (Oren)	100.0
12	Cloves (Bunga cengkih)	83.3	Pepper (Lada)	100.0	Cinnamon (Kayu manis)	90.0	Mustard (Mustard)	63.3
13	Pineapple (Nenas)	96.7	Pear (Buah pir)	80.0	Plum (Buah plum)	80.0	Peach (Buah pic)	83.3
14	Rose (Bunga ros)	96.7	Chamomile (Bunga chamomile)	33.3	Raspberry (Rasberi)	53.3	Cherry (Buah ceri)	80.0
15	Anise (Bunga lawang)	73.3	Rum (Arak)	NT	Honey (Madu)	100.0	Fir (Pokok cemara)	10.0
16	Fish (Ikan)	96.7	Bread (Roti)	100.0	Cheese (Keju)	100.0	Ham (Ham)	NT

Nine of the distractors were found to had lower identification rate of <70%, namely ‘buah beri hitam’ (43.4%), ‘daun kucai’ (50.0%), ‘pokok cemara’ (10.0%), ‘kacang walnut’ (66.7%), ‘limau gedang’ (63.3%), ‘mustard’ (63.3%), ‘acar kobis’ (63.3%), ‘bunga chamomile’ (33.3%), and ‘rasberi’ (53.3%). These problematic items were replaced by more familiar terms (Table 2). All the culturally modified odorants and distractors were retested for familiarity and achieved good percentage of >70%. 

**Table 2:** The familiarity percentage of the descriptors for odorants and distractors

**Original descriptor** **Malay English**		**Familiarity (%)**	**Replaced descriptor** **Malay English**		**Familiarity (%)**
1. Odorant (a) Licorice (b) Turpentin	Licorice Turpentine	13.3 36.7	Jintan manis Pencair cat ‘thinner’	Fennel seed Paint thinner	83.3 86.6
2. Distractors (a) Buah beri hitam (b) Daun kucai (c) Pokok cemara (d) Kacang walnut (e) Limau gedang (d) Mustard (e) Acar kobis (f) Bunga chamomile (g) Rasberi	Blackberry Chive Fir Walnut Grapefruit Mustard Sauerkraut Chamomile Raspberry	43.3 50.0 10.0 66.7 63.3 63.3 53.3 33.3 53.3	Durian Kunyit Daun pandan Rambutan Betik Halia Kunyit Bunga melur Cempedak	Durian Turmeric Pandan leaf Rambutan Papaya Ginger Turmeric Jasmine Jack fruit	100.0 96.7 100.0 93.3 96.7 96.7 96.7 93.3 100.0

 Three of the distractors were not tested, namely ‘*wain*’ (wine), ‘arak’ (rum) and *‘ham’ *(ham) considering the religion sensitivity in the multiracial local population especially to the majority Muslim community. The items were replaced with *‘petrol’ *(petrol), *‘*buah mangga’ (mango) and *‘*langsat’ (lanzones) respectively, and all achieved good familiarity percentage (100.0%, 100.0%, and 80.0% respectively). In summary, 16 items in the original version of SS-SIT were replaced, involving 12 cards. This includes 2 odorants descriptors, and 14 distractors (including 2 repetitive items *‘mustard’* and ‘pokok cemara’). The correct identification rate of each test odorants were 70.0 % - 100% after modification of the distractors (Table.3). 

**Table 3:** The correct identification rate of the modified list of the test odorants

**Card**	**The modified odorants**	**Correct identification rate (%)**
1	Oren	100.0
2	Beg kulit	70.0
3	Kayu manis	86.7
4	Pudina	100.0
5	Pisang	96.7
6	Lemon	73.3
7	Jintan manis	90.0
8	Pencair cat thinner	76.7
9	Bawang putih	86.7
10	Coffee	93.3
11	Epal	70.0
12	Bunga cengkih	83.3
13	Nenas	70.0
14	Bunga ros	76.7
15	Bunga lawang	96.7
16	Ikan	100.0

*Phase 3: Evaluation of construct validity using the cultural adapted version*

The mean smell identification score of the nosmosmic group [13.7 (1.1)] was significantly higher than the hypo-anosmic group [7.3 (3.4); t = 13.85 (df = 71.46), P <0.001]. 

*Phase 4: Evaluation of test-retest reliability* The similar test repeated two weeks apart showed consistent results, with the mean score of normosmic group [14.5 (0.96)] was significantly higher than the hypo-anosmic group [7.1 (2.67), t=20.31 (df=74.10), P<0.001] The correlation coefficient between test and retest score (r) was 0.93 (P<0.001), indicating high reliability of the adapted version. 

## Discussion

There is lack of usage of validated smell identification tool in local clinical practice generally. Sniffin’ Sticks smell identification test has long been validated in European and American countries, but lacking in Asian countries. Other than Taiwan, Korea and Sri Lanka, there is no study in Asian region on cultural adaptation of Sniffin’ Sticks smell identification test, including Malaysia.

Smell identification test is an important tool for assessment of olfactory function. However, the results greatly depend on the familiarity with the odorants, making its application difficult in different countries with different cultures. It is used in many countries but the results seem to be culture-dependent (3). The original version was developed in Germany based on odours familiar to Europe population. However, during cross-cultural application in other European countries, the SS-SIT appears to perform well in some but in many others it required adaptation using different descriptors for odorants and distractors. An Italian study on healthy volunteers showed good identification rates of all test odorants without modifications (12). Similarly, a study conducted in Australia had developed normative data for their population for Sniffin’ Sticks with no modification of the original version (9).

However, the olfactory test contains descriptors that are unfamiliar to Asians such as ‘sauerkraut’ and ‘mustard’, which may cause underestimation of olfactory function (22). Therefore in order to obtain a valid results, the odours tested and their verbal descriptors as well as the distractors should be adjusted to suit the subjects’ cultural and linguistic background. Cultural adaptation is a necessary action when adopting an evidence-based intervention or measuring tool with other ethnic groups. The process has been described in two parts, (i) the assessment of conceptual and linguistic equivalence, and (ii) the evaluation of measurement properties. Performance in the SIT relies on personal experience and familiarity of the presented odours (7). Each country and population has its own unique odour familiarity, which is dynamic and influenced by various factors such as food preparation, nutritional habits, different substances encountered in daily life, environmental variables such as geographic location and immigration (15,17). Therefore, the odorant used in a SIT must be highly familiar to the subject of the different cultural background. When applied cross-culturally, linguistic changes and replacement of the less familiar item is necessary. Since the test is based on a multiple forced-choice procedure, the descriptors for both the odorants and the distractors should be analysed and adapted before it can be administered. Non-adaptation of the SIT can produce a wrong diagnosis because of the unfamiliar items to the target population. 

Malaysia is a country consists of multiracial population with diverse cultural practices and language used. Majority the Malaysian people speaks a common language, the Bahasa Malaysia (Malay language), which is the national language. Lingusitic changes by translating the distractors to Bahasa Malaysia are therefore necessary to eliminate the difference in understanding and interpretation. 

Several methods have been employed in adaptation of SS-SIT in various studies of different cultural background. This includes change of descriptors of the odorant without changing the original odorant in the stick, replaced the odorant with a familiar one, modify the distractors list or combination of the above methods (10,14-17,19,20,22). 

From the study, we found that certain odorants were not recognised by local people partially because they were not familiar with the term used, for example ‘licorice’ and ‘turpentin’. Without having to change the odorant in the sticks, the correct identification rate had increased by changing the term to a more familiar one to local people, namely ‘licorice’ to ‘jintan manis’ and ‘turpentin’ to ‘pencair* cat *thinner’. Although ‘licorice’ and ‘jintan manis’ describe different plant, their smell is almost similar, largely due to a common organic compound called anethole that contributes to their odour and flavour. Similar findings were found in the Greek study, which showed low identification rate (<70%) for six of the odorants, namely ‘aniseed’, ‘turpentine’, ‘liquorice’, ‘apple’, ‘lemon’ and ‘cinnamon’ (10). Linguistic modifications of certain descriptors (e.g. ‘painter oil’ instead of ‘turpentine’, ‘Greek grappa’ instead of ‘licorice’) had significantly increased the identification of the problematic items. In a Romanian study three odorants with low identification percentage were replaced by names that are more familiar to their society, namely ‘licorice’ by ‘sweet root’, ‘turpentine’ by ‘dissolvent’ and ‘anis’ by ‘fennel’(15).

Studies in Asian populations found several unfamiliar odours with low identification rate such as ‘turpentine’, ‘anise’ and ‘cloves’ that were either renamed or replaced (19,20,22,25). In a validating study conducted in Taiwan, ‘turpentine’, ‘cloves’, and ‘anise’ were changed to ‘tiger balm’, ‘wood’, and ‘star anise’ respectively (20). These terms are more common and familiar to local population and indicate the same or similar odours. The authors concluded that the SS-SIT is suitable for the evaluation of olfactory function in an Asian population after modification of the descriptors.

It is important to modify the descriptors not only for the odorants but also the distractors using terms that are familiar to populations with different cultural background (15). 

A prospective study conducted in Hong Kong to assess olfaction in patients with nasopharyngeal carcinoma using Sniffin’ Sticks found that the mean identification score was 11.7 (2.41) before radiotherapy and 10.9 (2.66) 12 months after irradiation (25). Two of the odorants descriptors were replaced but the distractors were not revised. The patients were considered as normosmic pre-treatment but the mean score was lower than the 12.0, which indicate hyposmia. Therefore, this result may not reflect the true olfactory function of the patients. 

Out of all the distractors from the study, nine items had low familiarity of less than 70% (Table 2). This could be attributed to two factors. Firstly, some of the smells were culturally unfamiliar, such as ‘buah beri hitam’, ‘kacang walnut’, ‘acar kobis’, and ‘rasberi’. Secondly the respondents were unfamiliar to the terms used to described names of smells such as ‘daun kucai’, ‘limau gedang’, ‘bunga chamomile’, ‘pokok cemara’ and ‘mustard’. Identifiability of an odour depends on one’s past experience; the odour has to be encountered before and able to establish an association between the memory of the odour and its name (17).The respondents tend not to choose the item if they do not understand the meaning of the term or have no experience to the smell before. It is therefore important to remove these unfamiliar distractors and replace them with items that are more familiar to target population in order to increase the validity of the test. Modification of distractors list has been shown to be effective in increasing the identification rate of the odorants. A Turkish study revealed four odorants with low identification rate less than 75% (‘orange’, ‘turpentine’, ‘apple’ and ‘pineapple’), which increased after modifications of the distractors list. The authors proved that an odorant with low familiarity might achieve high identification rate by means of excluding other well-known distractors (17). This helps to safe and maintain the original odorants in the Sniffin’ Sticks battery. 

The identification rates for certain fruity odours such as ‘lemon’, ‘epal’ and ‘nenas’ were found to be relatively low (70.0% - 73.3%), possibly due to perceptual similarity of the distractors with the odorant (14). The use of more contrasted distractors in SIT can increase the identification rate of the odorant. In a randomised study the researchers modified the distractors list using more contrasted items, and they found that the correct identification was significantly increased in patients with hyposmia but not in patients with anosmia (26). In Romanian study, two of the problematic odorants with low identification percentage were kept the same (‘lemon’ and ‘apple’), but the distractors were changed to increase the contrast.^15^ For example ‘grapefruit’ to ‘menthol’, ‘apple’ to ‘onion’, ‘orange’ to ‘cheese’ and ‘peach’ to ‘cherry’. Study in Great Britain had proposed to modify the original list of distractors to more contrasted items (14). For example, ‘blackcurrant’, ‘strawberry’ and ‘vanilla’ as distractors for the odorant ‘apple’; ‘melon’, ‘peach’ and ‘orange’ as distractors for the odorant ‘lemon’. Similarly in the Taiwanese study, the familiarity percentage of ‘leather’, ‘cinnamon’ and ‘licorice’ were relatively low at 52-62% but the correct identification rates were high at 79-92% without having to change the descriptors (20). Estonian researchers modified the distractors so that the choice would be more easily made by exclusions, and the resultant increased in correct identification (27). This showed that the contrast of the distractors and exclusion strategy play a role in helping the respondents to choose the correct answer. 

Smell perception not only varies among different countries, but among different religions and cultural practices. Religious sensitivity and taboos should therefore be taken into account during cultural adaptation to avoid offence to the participants. Several items in the list that were deemed culturally irrelevant and inappropriate to be tested among Muslim subjects such as ‘wine’, ‘rum’ and ‘ham’ were omitted and replaced. 

Both the mean test and retest smell identification scores of the normosmic group were significantly higher than the hypo-anosmic group. This indicates that the test is capable of discriminating between healthy controls and patients with olfactory disorder. The test-retest correlation coefficient in this study was high (r = 0.93*)*. It is higher compared to other similar studies worldwide, such as 0.62 in Portugal, 0.73 in German, 0.78 in Romanian and 0.85 in Taiwan study (5,13,15,19). It is comparable with the result of the 40-item University of Pennsylvania Smell Identification Test (*r *= 0.92) (28). Our study has several limitations. The SS-SIT has its own disadvantage in which the methodology is based on multiple forced-choice odour selection. Patients with anosmia are likely to answer correctly by chance with a probability of 25% even though they cannot detect any odour. It is also difficult to differentiate hyposmia from malingering subjects by a low smell identification score. Although Malay is the major language spoken in Malaysia, other languages and dialects are also commonly used by the country’s large ethnic minorities. Some may not recognise the name of the odorants in language other than their mother tongue, which may cause bias in the results. 

## Conclusion

In conclusion, cultural adaptation is a prerequisite procedure before routine clinical use of the SS-SIT in the country. To our knowledge this is the first study in South East Asian region for cultural adaptation of SS-SIT. This study showed that the Malaysian version of SS-SIT is valid and has high test-retest reliability. It can be applied cross-culturally in Malaysia after precise translation and having some of the descriptors modified. It accurately reflects patients’ olfactory function and can be used as a screening test for smell dysfunction as well as an effective tool for olfactory function follow-up in our clinical practice. 

The SS-SIT can be coupled with the threshold and discrimination tests for a complete assessment of olfactory function. The validated Malaysian version enables further establishment of local normative data for clinical use (Table.4). This will provide a standardised evaluation of patients with olfactory dysfunction and permit comparison of clinical and research results obtained from different centres around the world. 

**Table 4:** The cultural adapted Malaysian version of Sniffin’ Sticks Smell Identification Test

**Card**	**Odorant**	**Distractor 1**	**Distractor 2**	**Distractor 3**
1	Oren	Durian	Strawberi	Nenas
2	Beg kulit	Asap	Gam	Rumput
3	Kayu manis	Madu	Vanila	Coklat
4	Pudina	Kunyit	Daun pandan	Bawang merah
5	Pisang	Kelapa	Rambutan	Buah ceri
6	Lemon	Buah pic	Epal	Betik
7	Jintan manis	Buah ceri	Pudina	Biskut
8	Pencair cat ‘thinner’	Halia	Getah	Mentol
9	Bawang putih	Bawang merah	Kunyit	Lobak
10	Kopi	Rokok	Petrol	Asap lilin
11	Epal	Tembikai	Buah pic	Oren
12	Bunga cengkih	Lada	Kayu manis	Halia
13	Nenas	Buah pir	Buah plum	Buah pic
14	Bunga ros	Bunga melur	Cempedak	Buah ceri
15	Bunga lawang	Buah mangga	Madu	Daun pandan
16	Ikan	Roti	Keju	Langsat

## Funding:

This study received grant from UKMMC Fundamental Research Fund (Research code: FF-2015-394).
